# Prevalence and distribution of musculoskeletal pain in patients with dizziness—A systematic review

**DOI:** 10.1002/pri.1941

**Published:** 2022-02-21

**Authors:** Unni Moen, Liv Heide Magnussen, Kjersti Thulin Wilhelmsen, Frederik Kragerud Goplen, Stein Helge Glad Nordahl, Dara Meldrum, Mari Kalland Knapstad

**Affiliations:** ^1^ Western Norway University of Applied Sciences Bergen Norway; ^2^ Norwegian National Advisory Unit on Vestibular Disorders Haukeland University Hospital Bergen Norway; ^3^ Department of Otorhinolaryngology & Head and Neck Surgery Haukeland University Hospital Bergen Norway; ^4^ Department of Clinical Medicine University of Bergen Bergen Norway; ^5^ The School of Medicine Trinity College Dublin The University of Dublin Dublin Ireland

**Keywords:** dizziness, musculoskeletal pain, vertigo

## Abstract

**Background and purpose:**

Musculoskeletal disorders are among the leading causes of disability globally, but their role in patients with dizziness and imbalance is not well understood or explored. Such knowledge may be important as musculoskeletal pain and dizziness can mutually influence each other, leading to a complex condition requiring more comprehensive approaches to promote successful recovery. We conducted a systematic review to examine the extent and characteristic of reported musculoskeletal pain in patients with dizziness.

**Methods:**

A comprehensive literature search in Medline, Embase, Cochrane, Scopus, Amed, Google Scholar, SveMed+, and Web of Science was conducted in March 2021. Inclusion criteria were studies examining patients with a vestibular diagnosis, patients with cervicogenic dizziness and patients included based on having dizziness as a symptom; and reported musculoskeletal pain. Data regarding age, sex, sample size, diagnosis and musculoskeletal pain was extracted. The Crowe Critical Appraisal Tool was used for assessing methodical quality of the included studies.

**Results:**

Out of 1507 screened studies, 16 studies met the inclusion criteria. The total sample consisted of 1144 individuals with dizziness. The frequency of patients reporting pain ranged between 43% and 100% in the included studies. Pain intensity were scored between 5 and 7 on a 0–10 scale. Pain in the neck and shoulder girdle was most often reported, but musculoskeletal pain in other parts of the body was also evident.

**Discussion:**

In the included studies, musculoskeletal pain was highly prevalent in patients with dizziness, with pain intensity that may have a moderate to severe interference with daily functioning. Pain in the neck and shoulder is well documented, but there are few studies addressing musculoskeletal pain in additional parts of the body. More research is needed to understand the relations between dizziness and musculoskeletal pain.

## 1 INTRODUCTION

Dizziness is a complaint with several potential etiologies, such as cardiovascular and neurological diseases, psychiatric disorders, metabolic disorders, infections, side effects from medications and vestibular disorders. It is a symptom often described as a feeling of vertigo, motion sensitivity, lightheadedness/presyncope, or disequilibrium/imbalance (Sloane et al., 2001; Sommerfeldt et al., 2021). In most cases dizziness passes quickly, but in 30%–50% of the cases the symptoms become persistent (Cousins et al., 2017; Godemann et al., 2005; Heinrichs et al., 2007; Kammerlind et al., 2005). Experiencing persistent dizziness can be exhausting and disabling with concomitant ailments such as musculoskeletal pain (Malmström et al., 2021), and psychological comorbidity (Lahmann et al., 2015), resulting in reduced quality of life and sick leave (Iglebekk et al., 2013; Neuhauser et al., 2008; Tjell et al., 2019). In some cases dizziness eventually results in long‐lasting conditions, such as persistent postural‐perceptual dizziness (PPPD) (Bittar & von Söhsten Lins, 2015).

Previous studies have found associations between dizziness and neck pain (Knapstad, Goplen, et al., 2019; Knapstad, Nordahl, et al., 2019; Malmström et al., 2007), where a possible explanation is that neck pain may cause dizziness via connections between the cervical proprioceptive system and the vestibular nuclei (Kristjansson & Treleaven, 2009; Peng, 2018). It could be advocated that the same rationale would apply for reduced somatosensory input from other parts of the body due to pain. Another explanation could be that patients “lock” the head to restrict head‐neck movements, to avoid triggering dizziness. This could lead to increased muscular tension in the neck/upper trunk area and pain may thus develop over time when dizziness persists (Kvåle et al., 2008; Wilhelmsen & Kvåle, 2014).

Some studies have reported that patients with cervicogenic dizziness (CD) (Malmström et al., 2007) and patients with peripheral vestibular dysfunction (Iglebekk et al., 2013; Kvåle et al., 2008; Malmström et al., 2021) also have pain in other parts of the body. The prevalence and distribution of musculoskeletal pain in patients with dizziness is uncertain. To further explore this is interesting since neck pain, which is frequently reported as a local phenomenon in this population (Knapstad, Goplen, et al., 2019), is often part of a wider pain pattern in the general population (Natvig et al., 2010).

The clinical picture in musculoskeletal disorders shows a striking overlap with what features can be found in disorders associated with dizziness, such as predominance in women (Neuhauser et al., 2005; Smith et al., 2014; Treaster & Burr, 2004; Yardley et al., 1998), increasing prevalence with increasing age (Sloane et al., 2001; Smith et al., 2014) and the common comorbidity of anxiety and depression (Blair et al., 2003; Cousins et al., 2017; Neuhauser et al., 2005). As there is a high prevalence of both musculoskeletal pain and dizziness in the general population, the chance of concomitant occurrence is high. We have argued that dizziness and pain can mutually influence each other negatively and lead to a complex condition that requires more comprehensive assessments and treatments to promote successful recovery. Thus, our aim was to conduct a systematic review to examine the extent and characteristic of reported musculoskeletal pain in patients with dizziness in the literature.

## 2 METHOD

### 2.1 Study design

We performed a systematic review employing the guidelines in the Preferred Reporting Items for Systematic Reviews and Meta‐Analyses (PRISMA) Statement (Moher et al., 2009; Page et al., 2021). The review study was registered in the Prospero database in advance of the data inclusion (CRD42020183285).

### 2.2 Search strategy

A comprehensive search in Medline, Embase, Cochrane, Scopus, Amed, Google Scholar, SveMed+, and Web of Science was conducted with assistance from an experienced librarian. The last search was accomplished in March 2021. The main search was targeting “dizziness” and “musculoskeletal pain” as MeSH terms in Medline, and further adjusted to the different databases. The search terms and full search strategy are presented in Appendix S1.

Both reviewers (UM and MKK) screened the reference list of the included studies and relevant review articles for potentially eligible studies not captured in the electronic search. Included studies are listed in Table 1.

**TABLE 1:** An overview of the included studies (*n* = 16) with sample size, age, gender, inclusion and exclusion criteria, main findings, and CCAT score

Study	Type of study	Study population	Inclusion criteria	Outcome measures	Main findings	CCAT score
Beh et al. (2019)	Retrospective	Vestibular migraine (VM) *n* = 131, (gender: 105 women [80.2%], 26 men; age: mean 44.3 ± 13.7)	Inclusion: Patients who fulfilled the 2012 International Headache society‐Bárány society. Criteria for VM Exclusion: Patients with potential overlap with other otologic and neurologic disorders	Retrospective chart review: Medical history and descriptions during most (>50%) of the VM attacks	Accompanying symptoms during VM attacks: 12/131 patients (9.2%) reported bilateral neck pain, 3 (2.3%) pain in arms and legs, 1 (0.8%) chest muscle pain. Self‐reported triggers of VM attacks: 3/131 patients (2.3%) reported neck pain as a trigger	30
Bjorne and Agerberg (2003)	Prospective	Ménière's disease *n* = 24 (gender: 14 women [58.3%], 10 men; age 29–74, mean age 52)	Inclusion: Consecutively chosen patients with definite (22) or suspected (2) Ménières disease	VAS (0–10) Custom‐made questionnaire	18/24 (75%) patients reported pain in the neck and shoulders VAS intensity approx. 7.2 12/24 (50%) patients reported pain in the face or jaws VAS intensity approx. 6.9	35
Bracher et al. (2000)	Prospective	Cervical vertigo (CV) *n* = 15 (gender: 12 women [80%], 3 men, age 27–82, median age 41)	Inclusion: Patients recruited from otorhinolaryngology practices with a primary complaint of dizziness and a working diagnosis of CV	Otorhinolaryng‐ology and chiropractic examination	14/15 (93%) patients reported musculoskeletal pain. 14/15 reported pain in the cervical region, shoulder‐girdle, or both. Of those, 7 (50%) reported pain in both regions. 7 patients with cervical pain reported primary pain in the suboccipital region. Duration of musculoskeletal pain varied from 80 days to 25 years, median duration 7.5 years.	27
Cuenca‐Martinez et al. (2018)	Observational, cross‐sectional	Cervicogen dizziness *n* = 64 (gender: 54 women [84.4%], 10 men: age 38–67, mean age 53)	Inclusion: Patients referred to the Physical Therapy Service of Primary care; age 18–70 years; chronic, non‐specific neck pain associated with CV > 3 months; presence of dizziness associated with pain, movements, stiffness, or specific postures of the cervical region	GCPS PPT	DHI lower group *n* = 32 (mean score 27.8): GCPS: 36,06 (SD 10.78); PPT: Trapezius 3.33 kg/cm^2^ (SD 2.29), suboccipital 2.92 kg/cm^2^ (SD 2.02) Months of pain: mean 42.03 VAS: Mean 64.1 DHI higher group *n* = 32 (mean score 56.75): GCPS: 48.72 (SD 12.13); PPT: Trapezius 3.69 kg/cm^2^ (SD 2.93), suboccipital 3.03 kg/cm^2^ (SD 2.55) Months of pain: mean 46.63 VAS: Mean 69.3 Lower DHI group statistically significant moderate and positive correlations were found between DHI and the magnitude of chronic pain (*p* < 0.01; *r* = 0.456) No significant correlations were found between the levels of disability due to dizziness and PPT in either of the two groups.	37
			Exclusion: Presence of neurological signs or other types or causes of dizziness, such as vertigo, vertebro‐basilar insufficiency, cardiovascular dizziness or migraines; or other causes of unsteadiness, patients who are in treatment or who have received some type of treatment in the last 3 months; communication difficulties			
Grande‐Alonso et al. (2018)	Cross‐sectional	Cervicogen dizziness and neck pain *n* = 20 (gender: 18 women [90%], 2 men; age 36.5 ± 11.03) Nonsymptomatic, no pain, no dizziness *n* = 22	Inclusion patients: Neck pain; neck disability; subjective feeling of dizziness associated with pain movement, rigidity or certain positions of the neck; neck pain and dizziness >3 months; 18–65 years Exclusion patient group: Trauma or recent surgery in head, face, neck or chest, an otorhinolaryngological diagnosis of central or peripheral vertigo, receiving physiotherapy during study period.	VAS (0–100)	VAS neck pain intensity = 62 (SD ± 14.4) No correlation was found between pain intensity and disability due to dizziness measured with DHI (*r* = 0.23)	36
Iglebekk et al. (2013)	Prospective, observational study	BPPV *n* = 69 (gender 49 women [71%], 20 men, age 21–68, median age 45	Inclusion: BPPV Exclusion: CNS disorder, migraine, eye movement ataxia, active Ménières disease, severe eye disorder	Symptom questionnaire (yes/no)	58 (87%) reported pain as a symptom. 58 patients (87%) reported neck pain, 27 (40%) reported widespread pain, 46 (67%) reported peri‐retroorbithal pressure/pain.	34
Knapstad, Nordahl, et al. (2019)	Prospective cross‐sectional	Dizziness only (DO) *n* = 100 (64% women, mean age 45.5) Dizziness with neck pain (DN) n = 138 (80%,3% women; mean age 45.7) Neck pain with dizziness (ND) n = 55 (83.6% women; mean age 42.5) healthy controls (HC) n = 47 (65%,9% women; mean age 40.5)	Inclusion: Persistent dizziness or neck pain, age 18–67 Exclusion: Language barrier, orthopedic or neurologic diseases known to affect balance, severe rheumatic disorders, vestibular schwannomas, and diving‐related inner ear disorders	PPT neck ACR	PPT upper neck: No statistically significant difference of PPT in HC compared to DO (27.7 kPa/cm^2^ vs. 25.6 kPa/cm^2^ [*p* = 0.83], DN group had statistically lower threshold compared to DO (19.0 kPa/cm^2^ vs. 25.6 kPa/cm^2^) (*p* < 0.001)	34
					PPT lower neck: HC had statistically significant higher PPT (25.7 kPa/cm^2^ vs. 21.2 kPa/cm^2^) compared to DO (*p* = 0.001), DN had significant lower threshold compared to DO (16.6 kPa/cm^2^ vs. 21.2 kPa/cm^2^ [*p*= < 0.001]) ACR tender points: No statistically significant difference in number of tender points in HC group compared to DO group (5.2 vs. 5.9) (*p* = 0.490). The DN group had statistically significant more tender points (11.6 vs. 5.9; *p* = < 0.001) compared to the DO group. 58% of the patients referred to ENT clinic had neck pain	
Krabak et al. (2000)	Retrospective, pilot study.	Dizziness with cervical origin *n* = 15 (11 women [73.3%], 4 men; age 23–76, mean 44.8)	Inclusion: Patients who had been treated over a 1‐year period with chronic cervical pain at a rehabilitative clinic Exclusion: Symptoms <1 year or history of trauma	Retrospective chart review	The patient had concurrent pain involving the Sternocleidomastoid (n = 11 [73%]), Trapezius (n = 10 [66%]), Levator scapulae (n = 9 (60%)), Occipitalis (n = 6 [40%]), Masseter (n = 3 [20%]), and/or Supraspinatus (n = 2 [13.3%]) VAS pain: mean 6,8 after a non‐standardized rehabilitation program focusing on cervical pain without balance or vestibular rehabilitation. Correlation between dizziness and pain (r = 0.58)	21
Kvåle et al. (2008)	Longitudinal design	Vestibular neuritis *n* = 32 (gender: 62.5% women; age 24–72, mean 48,3 years)	Inclusion: Uncompensated unilateral vestibular neuritis. Exclusion: Central vestibular disorder, progressive vestibular pathology, genetic hearing loss, neurological/visual/psychiatric disorders	Pain drawing (introduced after the project started *n* = 24)	Pain drawing, perceived pain last 14 days: 21/24 (87.5%) reported pain. 8 (33.3%) had pain localized to the upper body, 6 (25%) had pain only in the lower body and 7 (29%) had widespread pain. A total of 3 reported no pain.	33
Malmström et al. (2021)*	Cross‐sectional	Vestibular deficits and balance disorders *n* = 49 (gender: 34 women [69.4%], 15 men; mean age 52) Physically active older people *n* = 101	Inclusion: Referred to a tertiary referral for examination of vestibular deficits and balance disorders, able to understand and communicate, fill out questionnaires Exclusion: Failed to return questionnaire	Pain and dizziness questionnaire (custom made)	65%, 3% (SEM 6.9) presented co‐morbidity with pain to the levels that influenced daily life. Females suffered more often from pain than males (*p* = 0.013). Patients reporting pain scored significantly higher in the DHI total (*p* = 0.004) and the subscale DHI emotional (*p* < 0.001) and DHI functional (*p* = 0.011). Of those suffering from any kind of pain, the most common locations were: Neck/shoulders: 87%,5% Back: 65.5% Head: 56.3% Legs 43.8% A significant number of patients reporting pain had a history of an accident (*p* = 0.004).	32
Malmström et al. (2019)	Cross‐sectional	Balance and dizziness disorders *n* = 49 (gender: 34 women [69.4%], 15 men; mean age 52) Psychological population (*n* = 62) (only balance/dizziness population is targeted in this review)	Inclusion balance population: Referred from physicians to a vestibular unit for specialist examination of patients suffering from symptoms of dizziness and balance dysfunction	Custom made questionnaire detailing the properties of pain, dizziness with NRS scale 0–10	Balance/dizziness population: 61.2% reported NSB pain Pain duration: Mean 94 months NSB pain intensity 5.4 NSB pain severity 6.0 Frequency of reported pain in the balance/dizziness population: Neck/shoulder: 57% Back: 42% Head: 38% Legs: 29% Arms: 28% Feet: 15% Upper torso 12% Lower torso: 11% The balance/dizziness population reported significantly more often pain in the arms (*p* = 0.004) compared to the psychological population. Patients with dizziness (*n* = 85 from both groups) reported significantly more often pain in the head (*p* < 0.001), neck/shoulders (*p* < 0.001), and feet, compared with those who did not report dizziness.	35
Malmström et al. (2007)	RCT	Cervicogenic dizziness *n* = 22 at baseline (gender: 20 women (90.9%), 2 men; age: 25–49, mean age 37)	Inclusion: Simultaneous neck pain and dizziness, age <55 Exclusion: History of CNS disease, head‐neck trauma, major injuries in lower limbs, cerebrovascular diseases, ear diseases, psychiatric disorders, pregnancy, and hyperthyroidism	Duration of pain, palpation of 18 neck‐shoulder muscles (tenderness 0–3 scale, neck pain provoking test)	Bilateral tenderness was reported from the majority of the patients, especially in the dorsal neck and upper back muscles. Trapezius, suboccipital area, paraspinal or interscapular areas, and/or Levator Scapula was reported by >50% and had the highest scores of tenderness. The majority of patients had several tender zygapophysial joints, 11 patients in all levels, 4 at 5 levels, 3 at 3 levels, 2 at 2 levels, and 2 had no zygapophysial joint tenderness 19/22 had experienced pain more than 6 months, 17/22 had pain >12 months, 13/22 had pain >24 months, 9/22 had pain >60 months VAS intensity^a^ neck pain mean score 54 ± 23 in the first group and 56 ± 15 in the second group	32
Morinaka (2009)	Cross‐sectional	Cervical vertigo (CV) *n* = 176 (gender: 119 women (67.6%), 57 men; age: 24–91, median age 66 years)	Inclusion: CV based on the criteria of the Committee of the Japan Society for Equilibrium Research (1995) Exclusion: Not specified	CMI ‐questions about joint pain, pain of the upper or lower limbs, back pain, and lumbago	CMI: 75 patients (43%) (54 women, 21 men) ranged in age 29–91 years (median age 70 years) reported joint pain, pain in the upper or lower limbs, back pain, or lumbago. All 75 had at least one musculoskeletal disease Neck tenderness (sternocleidomastoid and along the nuchal line) was reported by 55 (31%) Tender areas in the sternocleidomastoid muscle and along the nuchal line were more than twice as frequent on the left side as on the right side	21
Reid et al. (2015)	RCT	Cervicogenic dizziness *n* = 86 (age 21–85, mean 62%; 50% female) randomized in three intervention/control groups.	Inclusion: Cervicogenic dizziness >3 months, history of neck pain Exclusion: Vertigo, psychogenic dizziness, vertebro‐basilar insufficiency, cardiovascular dizziness, migraine, or neurologic reasons for unsteadiness	VAS (0–100)	VAS intensity cervical pain: mean score of the three groups: 49.8	35
Thompson‐Harvey and Hain (2019)	Cross‐sectional	Cervical vertigo (CV) *n* = 16 (69% women, age 32–85), Vestibular migraine (VM) *n* = 16, (81% women, age 24–68), Vestibular vertigo (VV) *n* = 16, (56% women, age 37–82) Total *n* = 48 (age 24–85, mean 55)	Diagnosed by an expert in the field of otoneurology (history, imaging, vestibular and auditory tests)	Unique questionnaire (modified DHI and NDI) (*Two questions concerning pain)*	Head/neck/eye pain reported by 6 (37.5%) in VV group, 16 (100%) in CV group and 12 (75%) in VM group. A total 34 (70%) experienced pain in the head/neck/eye Neck pain or stiffness was reported by 5 (31%) in VV group, 15 (93%) in CV group and 11 (69%) in VM group. A total of 31 (65%) had pain or stiffness in the neck.	28
Tjell et al. (2019)	Retrospective	BPPV n = 163 (134 women [82.2%], 29 men; age 15–65, median age 43)	CVMCC diagnosis with history of trauma.	Structured Symptom Questionnaire (yes/no)	133 patients (82%) reported neck pain, 22 (13%) reported generalized pain. Peri‐/retroorbital press/pain was reported by 120 (74%) and temporo‐mandibular region pain reported by 82 (50%)	31

Abbreviations: ACR, The American College of Rheumatology's nine bilateral tender points; BPPV, Benign Paroxysmal positional vertigo; CMI, The Cornell Medical Index; CV, cervical vertigo; CVMCC, chronic vestibular multicanalicular canalithiasis; DN, dizziness and neck pain; DO, Dizziness only; GCPS, Graded Chronic Pain Scale; GPE‐52, Global Physiotherapy Examination—52; HC, healthy controls; NRS, Numeric Rating Scale; NSB, Neck‐Shoulder‐Back; PPT, pressure pain threshold; SEM, standard error of mean; VAS, Visual Analogue Scale; VM, vestibular migraine; VV, vestibular vertigo.

^a^ Baseline results originally published in the first study (Karlberg et al., 1996) on the same study population.

### 2.3 Eligibility criteria

This review was restricted to articles written in English or Scandinavian languages with no limitation to publication date. Published peer‐reviewed studies were included; unpublished studies, books, reviews, case reports, conference papers, editorials and papers not available in full text were not included. The inclusion criteria were studies examining participants with a vestibular diagnosis, patients with CD and patients included based on having dizziness as a symptom; and reported musculoskeletal pain. Regarding musculoskeletal pain, there were no restrictions concerning the type of outcome measures. To eliminate other types of pain, only studies that specified pain as musculoskeletal pain or referred to pain as pain in muscles, joints, bones or tendons were included. Studies concerning exclusively elderly >65 years were excluded to avoid bias as a result of natural changes due to aging. However, studies that included an adult population with a lifespan perspective were retained, even some in the sample were over 65 years of age.

### 2.4 Study selection

**FIGURE 1:**
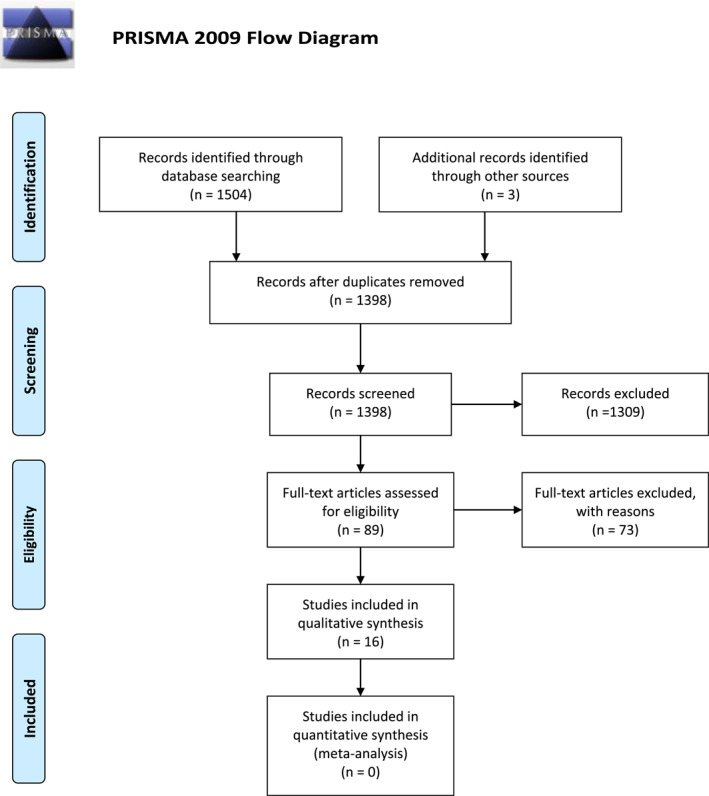
The PRISMA flow diagram details our search and selection process. *Source:* Moher et al. (2009)

After duplicates were removed, all titles and abstracts were screened independently by the two reviewers, removing all obvious irrelevant studies. Full‐text versions of eligible articles were evaluated by both reviewers for inclusion. Any disagreement was resolved through discussion between the reviewers and the co‐authors. The reviewer process was facilitated using Rayyan systematic review web application, which allowed blinding in each step of the process (Ouzzani et al., 2016). The selection process is documented in the PRISMA flowchart (Figure 1).

### 2.5 Data extraction

Bibliographic data (author, title, year, and study design), diagnosis, inclusion‐ and exclusion criteria, population (age and gender distribution), sample size, outcome measures regarding musculoskeletal pain were extracted, compared and compiled in a spreadsheet by both reviewers (Table 1). Only baseline information was extracted from intervention‐ or prospective studies to avoid bias due to treatment effect.

### 2.6 Data analysis

Due to the heterogeneous nature of the included studies concerning design and outcome measures, meta‐analyses of results were not feasible for this review. Thus, only a qualitative assessment of the data was conducted.

### 2.7 Quality assessment

The quality of the studies was assessed using the Crowe Critical Appraisal Tool 1.4 (CCAT; Crowe et al., 2012), which can be used across a variety of research designs. The CCAT Form is divided into eight categories, each category receives a score on a 6‐point scale (0–5). The maximum score is 40, where a higher score indicates higher quality. The total score is reported in Table 1.

### 2.8 Ethics

No ethical approvals were needed in this process. The authors state no conflict of interest.

## 3 RESULTS

### 3.1 Search result and study selection

The initial search identified 1507 articles. After duplicates were removed, 1398 references remained for the first screening. This first screening identified 89 studies for full‐text assessment, of which 73 did not meet the inclusion criteria, leaving 16 studies for qualitative analyses. The studies were published between 2000 and 2021.

Two of the included studies reported from the same study participants (Malmström et al., 2021; Malmström et al., 2019). This is highlighted in Table 1, and no information was duplicated in the analyses. The reason for exclusion is documented in Appendix S2.

### 3.2 Characteristics of included studies

#### 3.2.1 Demographics and diagnosis

The included studies comprised of 1144 unique individuals with dizziness, not counting controls. 73% of the study populations were women.

The studies included the following diagnoses: Two studies with vestibular migraine (Beh et al., 2019; Thompson‐Harvey & Hain, 2019), one study with Ménière's disease (Bjorne & Agerberg, 2003), two studies with benign paroxysmal positional vertigo (BPPV)/chronic vestibular multicanalicular canalithiasis (CVMCC; Iglebekk et al., 2013; Tjell et al., 2019), one study with vestibular neuritis (Kvåle et al., 2008), four with various dizziness diagnoses (Knapstad, Nordahl, et al., 2019; Malmström et al., 2021; Malmström et al., 2019; Thompson‐Harvey & Hain, 2019) and eight studies with CD (Bracher et al., 2000; Cuenca‐Martinez et al., 2018; Grande‐Alonso et al., 2018; Krabak et al., 2000; Malmström et al., 2007; Morinaka, 2009; Reid et al., 2015; Thompson‐Harvey & Hain, 2019).

#### 3.2.2 Methods for measuring pain

The outcome measures used to document the extent and intensity of musculoskeletal pain included Visual Analogue Scale (VAS), Numeric Rating Scale (NRS), Graded Chronic Pain Scale (GCPS), Pain Pressure Threshold (PPT), The American College of Rheumatology's (ACR) bilateral tender points, pain drawing, structured symptom questionnaires and chart reports.

VAS and NRS scores pain intensity on an 11‐point scale (0–10; NRS) or 0–100 mm (VAS). Both scales indicate 0 as “no pain at all” and 10 (100 mm) as “the worst pain ever possible”. GCPS consists of 8 items questioning pain intensity, duration of pain and interference of daily activities, social life and work, where 0 points equal “no pain”/“no interference” and 10 points equal pain as “bad as could be”/“unable to carry on any activities”.

### 3.3 Prevalence and location of musculoskeletal pain

The total prevalence of individuals reporting musculoskeletal pain was reported in 10 studies (Bjorne & Agerberg, 2003; Bracher et al., 2000; Iglebekk et al., 2013; Knapstad, Nordahl, et al., 2019; Kvåle et al., 2008; Malmström et al., 2019; Malmström et al., 2021; Morinaka, 2009; Thompson‐Harvey & Hain, 2019; Tjell et al., 2019), ranging between 43% and 100%.

### 3.4 Cervicogenic dizziness

In the studies with CD, four focused on pain in the neck, shoulder girdle and face (Bracher et al., 2000; Krabak et al., 2000; Malmström et al., 2007; Thompson‐Harvey & Hain, 2019), while one included pain in additional parts of the body (Morinaka, 2009). However, the diagnostic criteria for CD varied between the different studies.

In Bracher et al. (2000), 14 out of 15 (93%) patients reported pain in the neck and/or shoulder girdle, 47% had pain in both areas. Thompson‐Harvey & Hain (2019) found similar results, where 15 out of 16 patients (93%) reported pain or stiffness in neck, and 100% reported head/neck/eye pain. Krabak et al. (2000) found the Sternocleidomastoid muscle to be the most common site of pain in 73% of the patients (*n* = 15), followed by the Trapezius, Levator scapulae, Occipitalis, Masseter and Supraspinatus muscles. The same muscles were reported as painful in more than 50% of the individuals in the study by Malmström et al. (2007). In Morinaka (2009) 43% of the patients with cervical vertigo (*n* = 176) confirmed pain in the upper or lower limbs, joint pain, back pain or/and lumbago. Neck tenderness especially in the Sternocleidomastoid muscle and along the nuchal line, was the most painful area reported by 31% of the patients.

### 3.5 Oto‐vestibular diagnoses

Among 24 patients with Ménière's disease (Bjorne & Agerberg, 2003), 75% reported neck and shoulder pain, and 50% reported pain in the face or jaws. Similar results were found in two studies on BPPV (Iglebekk et al., 2013; Tjell et al., 2019) where 82%–87% reported neck pain, and 13% reported widespread/generalized pain. In the same studies, peri‐/retroorbital pain was reported by 67%–74%, and temporomandibular pain by 59%. In a study addressing clinical features and triggers of vestibular migraine (Beh et al., 2019), neck pain was reported as a trigger of attacks by 3 out of 131 patients. Twelve (9.2%) reported bilateral neck pain as an accompanying symptom during attacks, three had pain in the arms and legs and one had chest muscle pain during attacks. Thompson‐Harvey and Hain (2019) found that 12 out of 16 (75%) patients with vestibular migraine had pain in the head/neck/eye and 69% reported pain or stiffness in the neck. Among the patients with vestibular vertigo 37.5% (*n* = 16) reported head/neck/eye and 31% had pain or stiffness in neck. Pain in the head/neck/eye was significantly more frequent in vestibular migraine (*p* = 0.038) and cervical vertigo (*p* < 0.001) compared to vestibular vertigo. Neck pain/stiffness was significantly more frequent in cervical vertigo compared to vestibular vertigo (*p* < 0.001), but there was no significant difference (*p* = 0.093) compared to migraine patients. In Kvåle et al. (2008), 21 out of 24 patients with vestibular neuritis reported musculoskeletal pain. Using pain drawings, eight (33%) reported pain in the upper body, six (25%) reported pain in the lower body and seven (29%) reported both upper and lower body.

#### 3.5.1 Dizziness from various causes

Knapstad, Nordahl, et al. (2019) found that 58% of 238 patients referred to a specialized ear‐nose‐throat department due to dizziness and balance issues, reported neck pain. Malmström et al. (2021) found that 65.3% (*n* = 49) of patients referred to a vestibular unit suffering from dizziness and balance dysfunction, reported pain with severity that influenced daily life. In another study with the same participants, Malmström et al. (2019) reported that 61.2% had pain in neck, shoulder or back. Of these, pain was most often reported in the neck and shoulders (57%), followed by back pain (42%), legs (29%), arms (28%), feet (15%) and upper/lower torso (11%). In the patients with dizziness from both groups (*n* = 85), pain in the head, neck/shoulders and feet was reported significantly more often (*p* < 0.001), compared with those who did not report dizziness. Women suffered more often from pain than men (*p* = 0.013), and the patients reporting pain scored significantly higher in the Dizziness Handicap Inventory (DHI) total (*p* = 0.004) (Malmström et al., 2021).

### 3.6 Pain intensity

Reported VAS/NRS intensity varied from 5.0 to 7.2, an average score of 6.1 in total.

#### 3.6.1 Cervicogenic dizziness

The mean VAS score for neck pain in the five studies with CD was 6.0 (range: 5.0–6.9). Grande‐Alonso et al. (2018) examined pain intensity in 20 individuals and found a mean VAS score of 6.2. Malmström et al. (2007) studied a population in the age below 55 years (*n* = 22) and found an average intensity score of 5.5. Reid et al. (2015) (*n* = 86) reported an average VAS score of 5.0. Cuenca‐Martinez et al. (2018) reported a mean score of 6.4 in patients in the lower end of DHI (*n* = 32) and 6.9 in patients in the higher end of DHI (*n* = 32). Lastly, Krabak et al. (2000) examined 15 patients who had received a non‐standardized rehabilitation program for their neck pain over a 1‐year period in advance. The mean VAS score in this population was 6.8.

Cuenca‐Martinez et al. (2018) examined the magnitude of chronic pain evaluated with GCPS (*n* = 64). The patients in the higher end of DHI had statistically significant higher scores on the GCPS compared to those in the lower end, and the total mean score was 3.6.

#### 3.6.2 Vestibular diagnoses

Using the VAS scale, 24 patients with Ménière (Bjorne & Agerberg, 2003), scored 7.2 in the neck/shoulder and 6.9 for the face/jaw.

#### 3.6.3 Various dizziness diagnoses

Malmström et al. (2019) found that the mean VAS score of neck‐shoulder‐back pain intensity was 5.4, and the pain severity was 6.0 in patients with dizziness/balance disorders.

### 3.7 Muscle tenderness

#### 3.7.1 Cervicogenic dizziness

Malmström et al. (2007) examined muscle tenderness by palpation of 18 neck and shoulder sites. Tenderness was reported in the Trapezius muscle, suboccipital area, paraspinal areas, interscapular area and/or the Levator scapula muscle in >50% of the 22 subjects. Twenty patients (91%) had tender cervical zygapophyseal joints and of those, 11 reported tenderness at all cervical levels.

#### 3.7.2 Various dizziness diagnoses

Knapstad, Nordahl, et al. (2019) examined differences between dizzy patients, with and without neck pain. They found that patients with dizziness and no neck pain (*n* = 100) had lower PPT in the neck compared to healthy controls, but only in the lower neck area (C5–C6). The group with concurrent neck pain and dizziness (*n* = 138) had lower PPT compared to the dizzy group without neck pain in both the suboccipital area and the lower area of the neck. Further, the group with concurrent neck pain and dizziness reported more generalized pain (*p* < 0.001), measured with numbers of tender points (ACR) compared to the group without neck pain and the healthy controls. There was no statistical difference in the number of tender points in patients with dizziness without neck pain compared to healthy controls.

### 3.8 Duration of pain

Duration of musculoskeletal pain was described in four studies (Bracher et al., 2000; Cuenca‐Martinez et al., 2018; Malmström et al., 2007, 2019, 2021), all of them reported long‐lasting pain for at least 3 months.

#### 3.8.1 Cervicogenic dizziness

Bracher et al. (2000) reported a median duration of musculoskeletal pain of 7.5 years (range 80 days–25 years). Median duration of dizziness at baseline was 52 days (range 20–649 days). Malmström et al. (2007) found that nine out of 22 (41%) patients had experienced pain for more than 5 years, 59% had experienced pain for more than 2 years and 86% have had pain for at least 6 months Cuenca‐Martinez et al. (2018) reported an average duration of pain of approximately 3.5 years.

#### 3.8.2 Dizziness from various causes

Malmström et al. (2019) found that the mean duration of pain in patients with symptoms of dizziness and balance dysfunction (*n* = 49) was 7.8 years. Mean duration of dizziness was 5.8 years.

### 3.9 Quality of included studies

CCAT scores range from 21 to 37 indicating moderate to good quality. Common limitations were inadequate justification of sample size and sampling method, reporting of statistical analysis and insufficient information about ethical matters.

## 4 DISCUSSION

Approximately 70% of the individuals in the included studies reported musculoskeletal pain. Overall, pain was most often reported in the neck and shoulder areas, but this review showed that pain in other parts of the body was also evident. Muscles located along the nuchal line and shoulder girdle were the most prominent muscles related to pain, such as the Sternocleidomastoid, Trapezius, Levator scapulae, the suboccipital area, and the paraspinal‐ and interscapular muscles. Only four studies systematically examined pain in all parts of the body, thus, we still know little about the prevalence of musculoskeletal pain in other parts of the body. A high prevalence of neck pain in CD is not surprising as pain or cervical dysfunction is a central part of the diagnostic criteria. However, pain in the neck and shoulder girdle also seems to be common in patients with dizziness regardless of diagnosis (Bjorne & Agerberg, 2003; Iglebekk et al., 2013; Kvåle et al., 2008; Malmström et al., 2021; Tjell et al., 2019). Neck pain is a common musculoskeletal complaint in general, ranked as the fourth greatest contributor to global disability measured in years lived with disability (Hoy et al., 2014), yet the prevalence among the patients included in this review was higher (>50%) than in the general population (Hoy et al., 2010) (approximately 30%).

Iglebekk et al. (2013) found that pain in general was the second highest ranked symptom in chronic BPPV, and suggested a likely connection between neck pain, widespread pain and BPPV. The occurrence of pain in the two studies with vestibular migraine (Beh et al., 2019; Thompson‐Harvey & Hain, 2019) showed contradictory results. Beh et al. (2019) found that only a few patients reported pain as a trigger or accompanying symptom of migraine attacks, while Thompson‐Harvey and Hain (2019) reported neck pain and stiffness in over two‐thirds of the patients, examined with a modified Neck Disability Index. An explanation for this can be that Beh et al. (2019) was a retrospective chart review, pain was not a main outcome in the study, and only symptoms accompanying most (>50%) of the migraine attacks were reported. Thus, it is possible that pain was inadequately documented in the medical record.

The pain intensity/severity stated in the respective studies are consistent, reporting pain levels between 5 and 6 on a scale 0–10. According to the NRS cut‐off values for patients with chronic musculoskeletal pain (Boonstra et al., 2016), this reveals that pain overall has moderate to severe interference with functioning in daily life. An exception was seen in the study on patients with Ménière's disease who reported a slightly higher pain intensity in neck and shoulder (7.2), and face or jaw (6.9; Bjorne & Agerberg, 2003). A possible explanation may be the close relation between Ménière's and migraine (Radtke et al., 2002). An important note is that one study (Krabak et al., 2000) stated pain intensity *after* a 1‐year period of non‐standardized treatment for the cervical pain. The fact that the pain score in these patients remined high (6.8), emphasizes the substantiality of pain is in this population.

In the studies reporting the duration of pain, nearly all patients report pain for at least 3 months, which is classified as chronic pain (Treede et al., 2015). Previous studies have shown that chronic pain is a significant challenge in the general population, ranging between 12% and 34% in Europe (Breivik et al., 2006), but still not as common as reported among the individuals with dizziness in this review. Bracher et al. (2000) suggested that dizziness arises as an aggravating factor of chronic musculoskeletal dysfunction in the cervical spine and shoulder girdle, based on the marked difference between the average duration of vertigo (52 days) and the average duration of musculoskeletal symptoms (7.5 years).

### 4.1 Implications for clinical practice and research

Whether symptoms of dizziness and pain co‐exist independently of each other, or whether dizziness leads to muscle tenderness or vice versa, cannot be concluded based on our review. Our results do, however, indicate that it could be beneficial to assess and treat symptoms of pain as well as dizziness when pain is present in persistent dizziness.

Musculoskeletal pain seems to be highly prevalent in dizzy patients as indicated in our study, and it may be reasonable to ask whether assessment and treatment of pain need to be addressed routinely and more systematically than what is undertaken in clinical practice today. Increased knowledge of the extent, characteristics and distribution of musculoskeletal pain in patients with dizziness can broaden the perspective of treatment and understanding of long‐standing dizziness. The total burden of complaints could have a predictive value and inform expectations for recovery after treatment. Focusing on dizziness in isolation may overlook other important contributors to recovery and the patient's overall function.

### 4.2 Limitations

As dizziness occurs for many different reasons, a clear delimitation of inclusion criteria was challenging. We wanted to include patients suffering from dizziness where dizziness was the primary problem, excluding studies where dizziness may have been a symptom accompanying psychological, neurological or cardiovascular disorders, or along with natural aging or medications. CD and dizziness caused by whiplash or neck trauma may be overlapping etiologies, indicating potential bias in the inclusion process. However, as CD is viewed as a dizziness condition, we chose to include it in this review. The same argument was used for two of the papers including patients with chronic BPPV. One of the studies only included patients with a history of head trauma (Iglebekk et al., 2013) which would also likely be accompanied with pain from the musculoskeletal system. In addition to the fact that “chronic” BPPV is disputed, the patients were not diagnosed with BPPV according to international guidelines (von Brevern et al., 2015). However, we included the studies that primarily examined patients with dizziness.

The data on musculoskeletal pain was extracted regardless of the purpose of the study. The authors were aware of the risk of misrepresentation, and the result should therefore be interpreted with caution. Pain can originate from other sources than from the musculoskeletal system, but this was not discussed in any of the included studies. However, based on the context and the inclusion criteria in the separate studies, it is reasonable to assume that there was no other type of pain included in the studies. Studies with unclear definitions of pain, followed by missing or inadequate outcome measures of pain, were excluded. Further, the review reflects the prevalence of musculoskeletal pain in the included studies and does not necessarily reflect the prevalence of pain in all patients with dizziness.

## 5 CONCLUSION

Results from this review indicate that musculoskeletal pain affects a large proportion of individuals with dizziness disorders, with pain intensity that may have a moderate to severe interference with daily functioning. Duration and intensity of pain were comparable across the studies. Neck pain was prominent, not only in patients with CD but for dizziness disorders in general. Pain in other parts of the body was also evident, but the literature emphasizing this is scarce. The prevalence and intensity of musculoskeletal pain are valid for the included studies but cannot necessarily be generalized to all patients with dizziness. Further research to evaluate the association between musculoskeletal pain and dizziness is needed, also considering the characteristics of pain within the different diagnoses.

## CONFLICT OF INTERESTS

None.

## ETHICAL STATEMENT

No ethical approvals were needed.

## Supporting information

Supporting Information 1

Supporting Information 2

## Data Availability

The data that supports the findings of this study are available in the supplementary material of this article.
