# Supplementary material for: Prevalence and distribution of musculoskeletal pain in patients with dizziness—A systematic review
Source: Physiother Res Int. 2022 Feb 21;27(2):e1941. doi: 10.1002/pri.1941 (PMC9286866; doi:10.1002/pri.1941)
Supplement: Supplementary file 1 — Supporting Information 1 [file PRI-27-0-s002.docx]

Dokumentasjon av litteratursøk.

# Informasjon om søket.

| **Tittel på litteratursøk.** | Svimmelhet (PPPD) og muskelskjelettsmerter. |
| --- | --- |
| **Hvem har bestilt litteratursøket?** | Navn: Unni Moen  E-post: unni.moen@hvl.no |
| **Bibliotekar som har utført litteratursøket.** | Navn: Gøril Tvedten Jorem  Arbeidssted: HVL, Biblioteket Bergen  E-post: goril.tvedten.jorem@hvl.no  Tlf: 55 58 78 09 |

##### Systematiske oversikter

| **Database/kilde** | Cochrane |
| --- | --- |
| **Dato for søk** | 28.04.2020 |
| **Søkehistorie eller fremgangsmåte** | 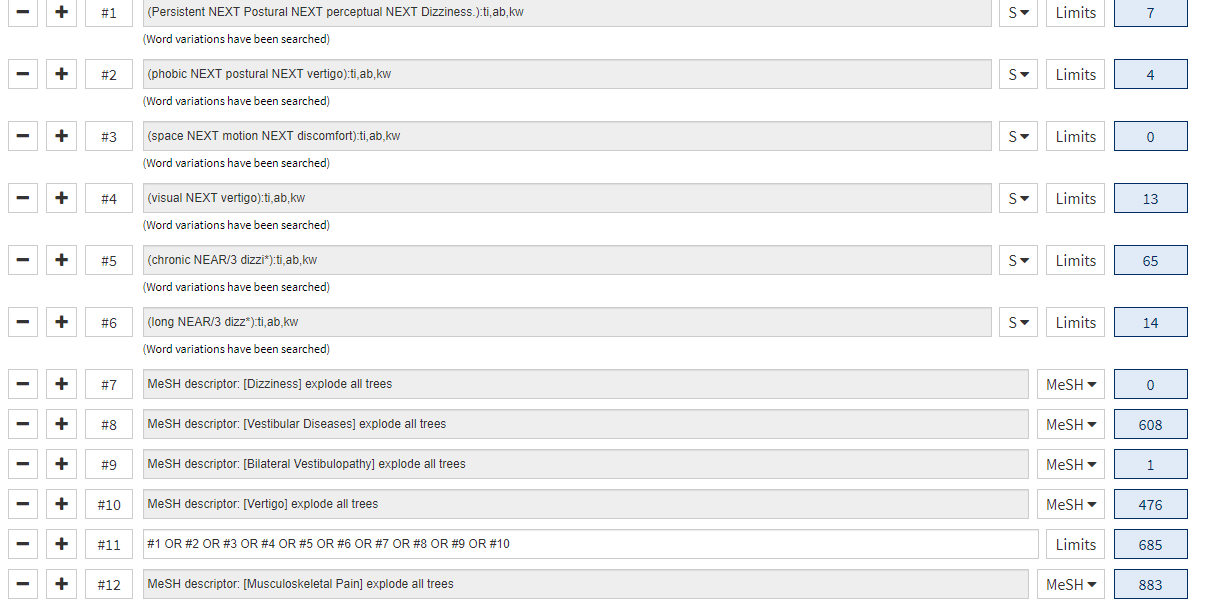  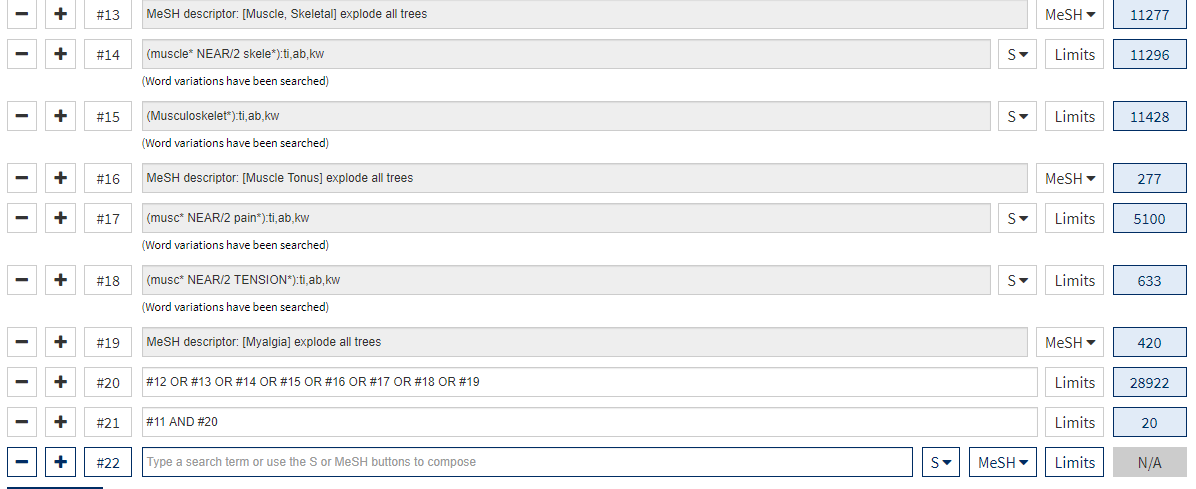 |
| **Antall treff** | 20 |
| **Kommentarer** |  |

##### Primærstudier

| **Database/kilde** | Medline |
| --- | --- |
| **Dato for søk** | 28.04.2020 |
| **Søkehistorie eller fremgangsmåte** | Database: Ovid MEDLINE(R) and Epub Ahead of Print, In-Process & Other Non-Indexed Citations and Daily <1946 to April 24, 2020>  Search Strategy:  --------------------------------------------------------------------------------  1 pppd.ti,ab,kw. (433)  2 Persistent Postural perceptual Dizziness.ti,ab,kw. (58)  3 (phobic adj postural adj vertigo).ti,ab,kw. (60)  4 (space adj motion adj discomfort).ti,ab,kw. (3)  5 (visual adj vertigo).ti,ab,kw. (58)  6 (chronic adj subjective adj dizziness).ti,ab,kw. (44)  7 (chronic adj dizzi*).ti,ab,kw. (164)  8 (chronic adj1 functional adj1 dizziness).ti,ab,kw. (1)  9 (long adj3 dizz*).ti,ab,kw. (39)  10 exp Dizziness/ (5261)  11 vestibular diseases/ or bilateral vestibulopathy/ or vertigo/ (13693)  12 or/1-11 (17997)  13 exp Musculoskeletal Pain/ (4914)  14 exp Muscle, Skeletal/ (264579)  15 (muscle* adj2 skele*).ti,ab,kw. (113823)  16 Musculoskelet*.ti,ab,kw. (50685)  17 exp Muscle Tonus/ (6711)  18 (musc* adj2 (pain* or tension*)).ti,ab,tw. (17089)  19 exp Myalgia/ (1655)  20 or/13-19 (386236)  21 12 and 20 (321)  22 exp Anxiety/ (83635)  23 anxiet*.ti,ab,kw. (189532)  24 stress*.ti,ab,kw. (821107)  25 22 or 23 or 24 (995599)  26 21 not 25 (298)  *************************** |
| **Antall treff** | 298 |
| **Kommentarer** |  |

| **Database/kilde** | Embase |
| --- | --- |
| **Dato for søk** | 28.04.2020 |
| **Søkehistorie eller fremgangsmåte** | Database: Embase <1980 to 2020 Week 17>  Search Strategy:  --------------------------------------------------------------------------------  1 (Persistent adj Postural adj perceptual adj Dizziness).ti,ab,kw. (68)  2 (phobic adj postural adj vertigo).ti,ab,kw. (110)  3 (space adj motion adj discomfort).ti,ab,kw. (3)  4 (visual adj vertigo).ti,ab,kw. (84)  5 (chronic adj3 dizzi*).ti,ab,kw. (375)  6 (long adj3 dizz*).ti,ab,kw. (63)  7 *dizziness/ (2126)  8 *vestibular disorder/ (4522)  9 *bilateral vestibulopathy/ (124)  10 *vertigo/ (10061)  11 or/1-10 (16115)  12 *skeletal muscle/ (42526)  13 *musculoskeletal pain/ (2852)  14 (muscle* adj2 skele*).ti,ab,kw. (132503)  15 Musculoskelet*.ti,ab,kw. (70848)  16 *muscle tone/ (2346)  17 *myalgia/ (4046)  18 (musc* adj2 (pain* or tension*)).ti,ab,tw. (23685)  19 or/12-18 (232887)  20 11 and 19 (229)  21 exp anxiety/ (197979)  22 anxiet*.ti,ab,kw. (269242)  23 exp stress/ (290495)  24 anxiet*.ti,ab,kw. (269242)  25 or/21-24 (570211)  26 20 not 25 (209) |
| **Antall treff** | 209 |
| **Kommentarer** |  |

| **Database/kilde** | Amed |
| --- | --- |
| **Dato for søk** | 29.04.2020 |
| **Søkehistorie eller fremgangsmåte** | Database: AMED (Allied and Complementary Medicine) <1985 to April 2020>  Search Strategy:  --------------------------------------------------------------------------------  1 pppd.ti,ab. (0)  2 Persistent Postural perceptual Dizziness.ti,ab. (0)  3 (phobic adj postural adj vertigo).ti,ab. (0)  4 (space adj motion adj discomfort).ti,ab. (0)  5 (visual adj vertigo).ti,ab. (2)  6 (chronic adj3 dizziness).ti,ab. (14)  7 (long adj3 dizz*).ti,ab. (5)  8 exp Dizziness/ (187)  9 exp Vestibular disease/ (313)  10 (bilateral adj vestibulopath*).ti,ab. (2)  11 exp Vertigo/ (164)  12 or/1-11 (451)  13 exp Musculoskeletal Pain/ (234)  14 exp Muscle skeletal/ (6523)  15 (muscle* adj2 skele*).ti,ab. (1221)  16 Musculoskelet*.ti,ab. (4084)  17 exp Muscle tonus/ (150)  18 (musc* adj2 (pain* or tension*)).ti,ab. (1770)  19 or/13-18 (11919)  20 12 and 19 (10)  *************************** |
| **Antall treff** | 10 |
| **Kommentarer** |  |

| **Database/kilde** | Google Scholar |
| --- | --- |
| **Dato for søk** | 29.04.2020 |
| **Søkehistorie eller fremgangsmåte** | "Persistent Postural perceptual Dizziness" "Musculoskeletal Pain"  "Persistent Postural perceptual Dizziness" ""muscle pain"  "Persistent Postural perceptual Dizziness" "muscle tension”  "pppd" "muscle tension"  "pppd" "muscle pain”  “pppd” “Musculoskeletal Pain”  "long* dizziness" “Musculoskeletal Pain”  "long* dizziness" “Muscle Pain”  "phobic postural vertigo" “Muscle pain”  "phobic postural vertigo" “Muscle tension”  "phobic postural vertigo" “Musculoskeletal Pain”  "space motion discomfort" “Musculoskeletal Pain”  "space motion discomfort" “muscle pain”  "space motion discomfort" “muscle tension*  "chronic subjective dizziness" “muscle tension”  "chronic subjective dizziness" “muscle pain"  "chronic subjective dizziness" “Musculoskeletal Pain”  "chronic dizziness" “muscle pain”  "chronic dizziness" “muscle tension"  "chronic dizziness" “Musculoskeletal Pain” |
| **Antall treff** | 322 |
| **Kommentarer** |  |

| **Database/kilde** | SveMed+ |
| --- | --- |
| **Dato for søk** | 28.04.2020 |
| **Søkehistorie eller fremgangsmåte** | 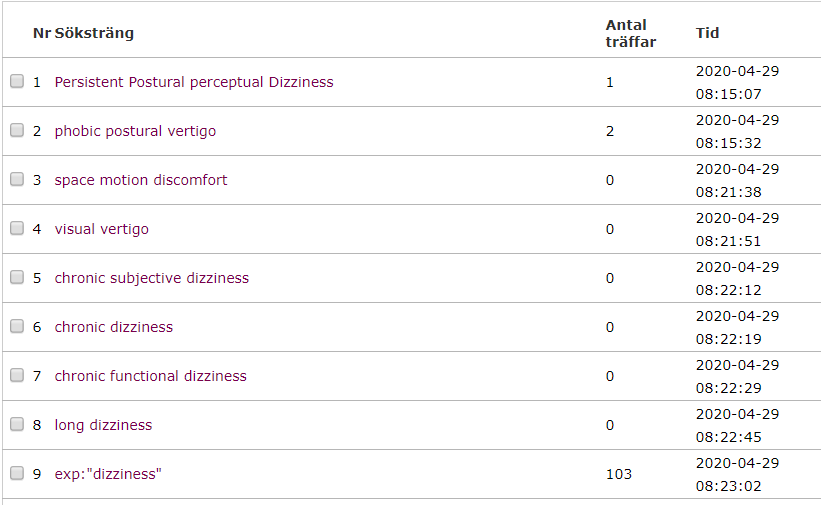  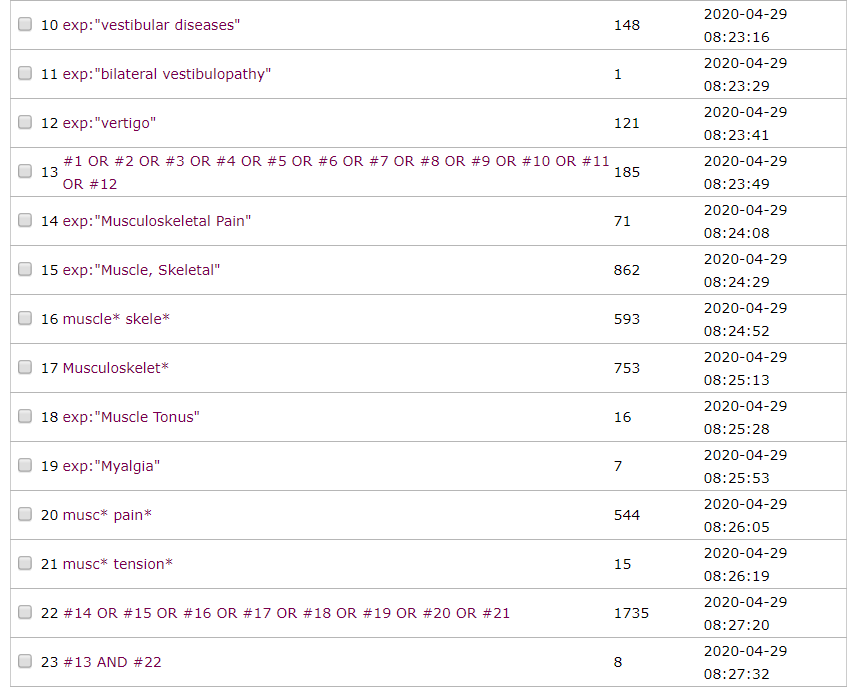 |
| **Antall treff** | 8 |
| **Kommentarer** |  |

| **Database/kilde** | Web of Science |
| --- | --- |
| **Dato for søk** | 28.04.2020 |
| **Søkehistorie eller fremgangsmåte** | 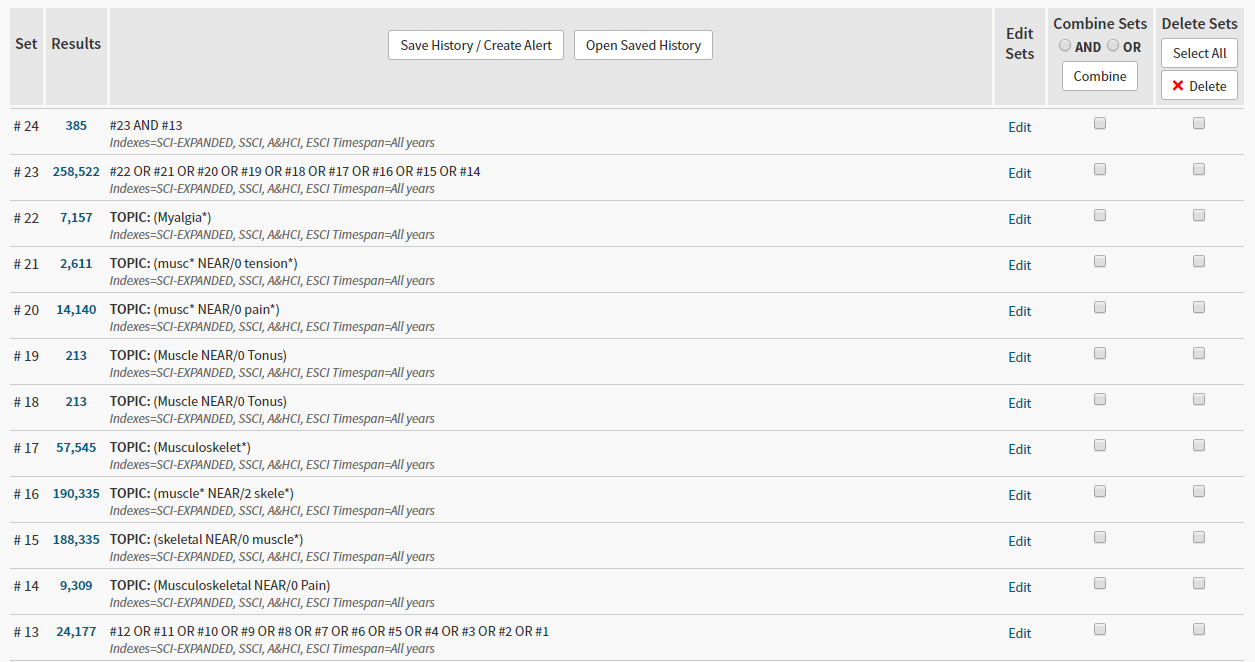  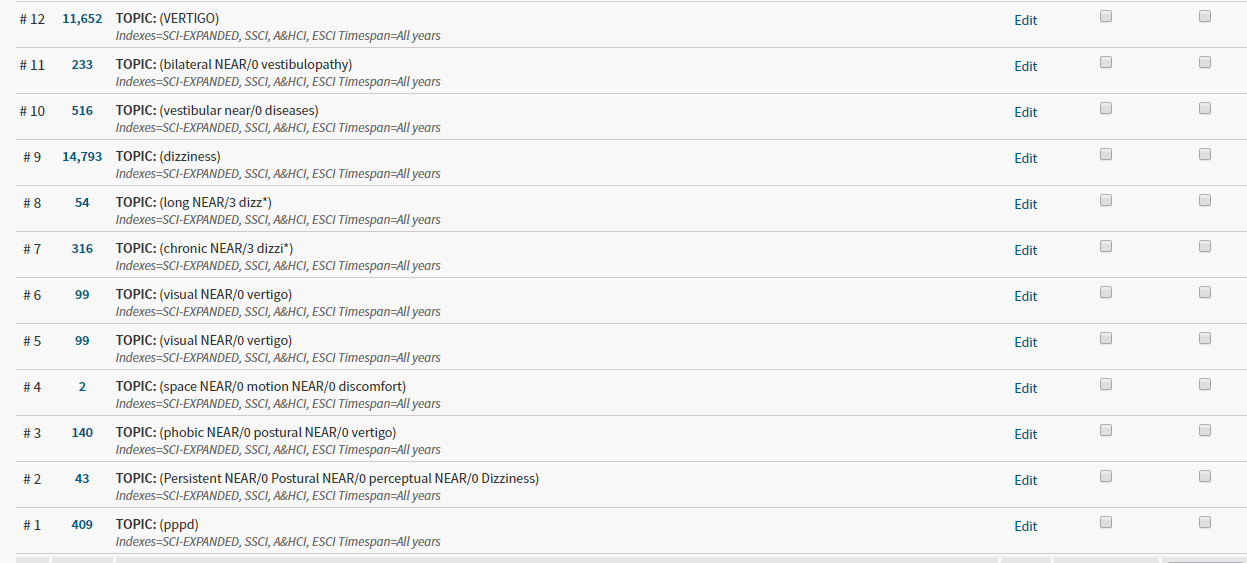 |
| **Antall treff** | 385 |
| **Kommentarer** | ikke mulig å fjerne stress og anxiety (ikke mulig å NOT’e). |
