# Supplementary material for: Prevalence and distribution of musculoskeletal pain in patients with dizziness—A systematic review
Source: Physiother Res Int. 2022 Feb 21;27(2):e1941. doi: 10.1002/pri.1941 (PMC9286866; doi:10.1002/pri.1941)
Supplement: Supplementary file 2 — Supporting Information 2 [file PRI-27-0-s001.docx]

**Appendix B.** Excluded studies with reason

| Publication | Reason for exclusion |
| --- | --- |
| Anonymous (2012) | Publication type: Magazine article |
| Aharoni, Goldman, Lubetzky, Krasovsky, and Zhu (2019) | Publication type: Conference paper |
| Alahmari et al. (2014) | No pain measures |
| Aratani, Ricci, Caovilla, and Grananca (2020) | No pain measures, wrong population: age ≥ 65 years |
| Asama, Goto, Tsutsumi, and Ogawa (2012) | No pain measures |
| Aydin, Dernek, Senturk Ege, Karan, and Aksoy (2018) | Wrong population: Cervical myofascial pain syndrom |
| Baloh (2021) | Publication type: Book |
| Benson, Gedye, and Jones (1963) | Full text not avaliable |
| Bittar and von Söhsten Lins (2015) | No pain measures |
| Borello-France, Gallagher, Furman, Redfern, and Carvell (2002) | No pain measures |
| Breivik (2015) | Publication type: Editorial comment |
| Bronstein, Lempert, and Seemungal (2010) | Study design: Review |
| Carvalho et al. (2020) | Study design: Review |
| Clark et al. (2000) | No pain measures |
| Clinical Trials (2016) | Publication type: Registered study, not published |
| Dietzek et al. (2018) | No pain measures |
| Eren et al. (2018) | No pain measures |
| Escaloni, Butts, and Dunning (2018) | Study design: Review |
| Grgic (2006) | Study design: Review |
| Habs, Strobl, Grill, Dieterich, and Becker-Bense (2020) | No pain measures |
| Haukanes (2020) | Publication type: Master thesis |
| Hauser | Publication type: Web page |
| Iglebekk, Tjell, and Borenstein (2015) | Wrong population: Musculoskeletal disorder |
| J. M. Holmberg (2020) | Study design: Review |
| J. Holmberg, Karlberg, Harlacher, Rivano-Fischer, and Magnusson (2006) | No pain measures |
| J. Holmberg, Karlberg, Harlacher, and Magnusson (2007) | No pain measures |
| Júnior et al. (2010) | No pain measures |
| Karlberg, Magnusson, Malmström, Melander, and Moritz (1996) | Population: Patients with neck pain |
| Knapstad, Nordahl, and Goplen (2019) | Study design: Review |
| Kollén, Hörder, Möller, and Frändin (2017) | Population: age = 75 |
| Kristiansen et al. (2019) | No pain measures |
| Kuwabara, Kondo, Kabaya, and Watanabe (2020) | No pain measures |
| Kwon and Ko (2017) | Study design: Review |
| Liu et al. (2017) | No pain measures |
| Lopes, Lemos, Figueiredo, and Santos (2019) | No pain measures |
| Malaktaris, Lemons, Lynn, and Condons (2014) | Publication type: Book |
| Malmström, Karlberg, Fransson, Lindbladh, and Magnusson (2009) | No pain measures |
| Meldrum et al. (2015) | No pain measures |
| Murdin and Harrop-Griffiths (2021) | Study design: Review |
| Nada, Ibraheem, and Hassaan (2019) | No pain measures |
| Odderson (2020) | Study design: Case report. No pain measures |
| Odkvist and Odkvist (1988) | Study design: Review |
| Pereira et al. (2020) | No pain measures |
| Piker, Jacobson, and Newman (2020) | Publication type: Book |
| Reid, Callister, Katekar, and Rivett (2014) | No pain measures |
| Ricci, Aratani, Caovilla, and Ganança (2012) | Publication type: Study protocol |
| Saman et al. (2016) | No pain measures |
| Schenk, Coons, Bennett, and Huijbregts (2006) | Study design: Case report |
| Scherer (1985) | Foreign language |
| Schmid et al. (2018) | No pain measures |
| Sezier, Saywell, Terry, Taylor, and Kayes (2019) | No pain measures |
| Sezier (2016) | Publication type: Master thesis |
| Stone and Carson (2017) | Publication type: Book |
| Sveinsdóttir (2014) | Publication type: Master thesis |
| Szirmai et al. (2020) | Foreign language |
| Talewar, Cassidy, and McIntyre (2018) | No pain measures |
| Z. Thomas and Smith (2020) | Publication type: Poster |
| L. Thomas and Treleaven (2020) | No pain measures; publication type: professional issue |
| Thoomes-de Graaf and Thoomes (2016) | Publication type: Book |
| Tjell and Iglebekk (2012) | Publication type: Book |
| Tjernström, Zur, and Jahn (2016) | Study design: Review |
| Tramontano, Consorti, Morone, and Lunghi (2020) | Study design: Review |
| van de Berg et al. (2021) | Population: children |
| Walker, Kantaris, and Mary (2018) | No pain measures |
| Weeks and Travell (1955) | Full text not available |
| Whitney, Sparto, and Furman (2020) | Study design: Review |
| Wilhelmsen (2010) | Publication type: Doctoral thesis |
| Wilhelmsen and Kvåle (2014) | Study design: Case series |
| Wurthmann et al. (2021) | No pain measures |
| Yao et al. (2020) | No pain measures |
| Zaleski-King (2020) | Publication type: Dissertation |
| Zhang, Liu, and Fu (2014) | Foreign language |
| Zur et al. (2015) | No pain measures |

Aharoni, M. M. H., Goldman, M., Lubetzky, A. V., Krasovsky, T., & Zhu, W. (2019). *A Virtual Reality Four-Square Step Test for Quantifying Dynamic Balance Performance in People with Persistent Postural Perceptual Dizziness.* Paper presented at the Proceedings of the 13th International Conference on Virtual Rehabilitation (ICVR), Tel Aviv.

Alahmari, K. A., Sparto, P. J., Marchetti, G. F., Redfern, M. S., Furman, J. M., & Whitney, S. L. (2014). Comparison of virtual reality based therapy with customized vestibular physical therapy for the treatment of vestibular disorders. *IEEE Transactions on Neural Systems & Rehabilitation Engineering, 22*(2), 389-399. doi:<https://doi.org/10.1109/TNSRE.2013.2294904>

Anonymous. (2012). A delicate balance: managing vertigo in general practice *Best Practice Journal(46)*. Retrieved from <https://bpac.org.nz/bpj/2012/september/docs/bpj_46_sep2012.pdf>

Aratani, M. C., Ricci, N. A., Caovilla, H. h., & Grananca, F. (2020). Benefits of vestibular rehabilitation on patient-reported outcomes in older adults with vestibular disorders: a randomized clinical trial. *Brazilian Journal of Physical Therapy*. doi:<https://doi.org/10.1016/j.bjpt.2019.12.003>

Asama, Y., Goto, F., Tsutsumi, T., & Ogawa, K. (2012). Objective evaluation of neck muscle tension and static balance in patients with chronic dizziness. *Acta Oto-Laryngologica, 132*(11), 1168-1171. doi:<https://doi-org.galanga.hvl.no/10.3109/00016489.2012.699197>

Aydin, T., Dernek, B., Senturk Ege, T., Karan, A., & Aksoy, C. (2018). The Effectiveness of Dry Needling and Exercise Therapy in Patients with Dizziness Caused By Cervical Myofascial Pain Syndrome; Prospective Randomized Clinical Study. *Pain Medicine, 20*(1), 153-160. doi:<https://doi-org.galanga.hvl.no/10.1093/pm/pny072>

Baloh, R. W. (2021). Chronic Dizziness. *Medically Unexplained Symptoms*. Retrieved from <https://link.springer.com/chapter/10.1007/978-3-030-59181-6_9>

Benson, A. J., Gedye, J. L., & Jones, G. M. (1963). Disorientation in flight due to a covert vestibular disorder, with associated generalised, muscular tension. *Aerospace Medicine, 34*, 649-654.

Bittar, R. S. M., & von Söhsten Lins, E. M. D. (2015). Clinical characteristics of patients with persistent postural-perceptual dizziness (PPPD). *Brazilian Journal of Otorhinolaryngology, 81*(3), 276-282. doi:<https://doi.org/10.1016/j.bjorl.2014.08.012>

Borello-France, D. F., Gallagher, J. D., Furman, J. M., Redfern, M. S., & Carvell, G. E. (2002). Voluntary upper-extremity movements in patients with unilateral peripheral vestibular hypofunction. *Physical Therapy, 82*(3), 216-227. doi:<https://doi.org/10.1093/ptj/82.3.216>

Breivik, H. (2015). An overlooked cause of head- and neck-pain: Chronic canalithiasis, or Benign Paroxysmal Positional Vertigo - BPPV. *Scandinavian Journal of Pain, 8*(1), 8-9. doi:<https://doi.org/10.1016/j.sjpain.2015.02.005>

Bronstein, A. M., Lempert, T., & Seemungal, S. B. (2010). Chronic dizziness: a practical approach. *Practical neurology, 10*, 129-139. doi:<http://dx.doi.org/10.1136/jnnp.2010.211607>

Carvalho, G. F., Schwarz, A., Szikszay, T. M., Adamczyk, W. M., Bevilaqua-Grossi, D., & Luedtke, K. (2020). Physical therapy and migraine: musculoskeletal and balance dysfunctions and their relevance for clinical practice. *Brazilian Journal of Physical Therapy, 24*(4), 306-317. doi:<https://doi.org/10.1016/j.bjpt.2019.11.001>

Clark, M. R., Heinberg, L. J., Haythornthwaite, J. A., Quatrano-Piacentini, A., Pappagallo, M., & Traja, S., N. (2000). Psychiatric symptoms and distress differ between patients with postherpetic neuralgia and peripheral vestibular disease. *Journal of Psychosomatic Research, 48*(1). doi:<https://doi.org/10.1016/S0022-3999(99)00076-8>

Clinical Trials. (2016). Assessment and Treatment of Patients With Long-term Dizziness in Primary Care. Retrieved from <https://clinicaltrials.gov/show/NCT02655575>. <https://clinicaltrials.gov/show/NCT02655575>

Dietzek, M., Finn, S., Karvouniari, P., Zeller, M., Klinger, C. M., Guntinas-Lichius, O., . . . Axer, H. (2018). In older patients treated for dizziness and vertigo in multimodal rehabilitation somatic deficits prevail while anxiety plays a minor role compared to young and middle afed patients. *Frontiers in Aging Neuroscience*. doi:<https://doi.org/10.3389/fnagi.2018.00345>

Eren, O., Filippopulos, F., Sönmez, K., Möhwald, K., Straube, A., & Schöberl, F. (2018). Non-invasive vagus nerve stimulation significantly improves quality of life in patients with persistent postural-perceptual dizziness. *Journal of Neurology, 265*, 63-69. Retrieved from <https://link.springer.com/article/10.1007/s00415-018-8894-8>

Escaloni, J., Butts, R., & Dunning, J. (2018). The use of dry needling as a diagnostic tool and clinical treatment for cervicogenic dizziness: a narrative review & case series. *Journal of Bodywork & Movement Therapies, 22*(4), 947-955. doi:<http://doi.org/10.1016>

Grgic, V. (2006). Cervicogenic proprioceptive vertigo: etiopathogenesis, clinical manifestations, diagnosis and therapy with special emphasis on manual therapy. *Lijecnicki Vjesnik, 128*(9-10), 288-295.

Habs, M., Strobl, R., Grill, E., Dieterich, M., & Becker-Bense, S. (2020). Primary or secondary chronic functional dizziness: does it make a difference? A DizzyReg study in 356 patients. *Journal of Neurology, 267*. doi:<https://doi.org/10.1007/s00415-020-10150-9>

Haukanes, L. (2020). *Samanhengen mellom muskelskjelettfunksjon og balanse hos pasientar med langvarig svimmelheit–Ein tverrsnittstudie.* (Master). Western Norway University of Applied Sciences, Bergen, Norway. Retrieved from <https://hvlopen.brage.unit.no/hvlopen-xmlui/handle/11250/2660976>

Hauser, R. Cervical Vertigo and Cervicogenic Dizziness. *caringmedical.com.* Retrieved from <https://www.caringmedical.com/prolotherapy-news/vertigo-treatment/>

Holmberg, J., Karlberg, M., Harlacher, U., & Magnusson, M. (2007). One-year follow-up of cognitive behavioral therapy for phobic postural vertigo. *Journal of Neurology, 254*(9), 1189. doi:<http://doi.org/10.1007/s00415-007-0499-6>

Holmberg, J., Karlberg, M., Harlacher, U., Rivano-Fischer, M., & Magnusson, M. (2006). Treatment of phobic postural vertigo. *Journal of Neurology, 253*, 500-506. doi:<https://doi.org/10.1007/s00415-005-0050-6>

Holmberg, J. M. (2020). Pathophysiology, Differential Diagnosis, and Management of Persistent Postural-Perceptual Dizziness: A Review. *Perspectives of the ASHA Special Interest Groups, 5*(1). doi:<https://doi.org/10.1044/2019_PERSP-19-00105>

Iglebekk, W., Tjell, C., & Borenstein, P. (2015). Treatment of chronic canalithiasis can be beneficial for patients with vertigo/dizziness and chronic musculoskeletal pain, including whiplash related pain. *Scandinavian Journal of Pain, 8*, 1-7. doi:<http://doi.org/10.1016/j.sjpain.2015.02.002>

Júnior, A. N. C., Gazzola, J. M., Gabilan, Y. P. L., Mazzetti, K. R., Perracini, M. R., & Ganança, F. F. (2010). Head and shoulder alignment among patients with unilateral vestibular hypofunction. *Brazilian Journal of Physical Therapy, 14*(4), 330-336. doi:<https://doi.org/10.1590/S1413-35552010005000022>

Karlberg, M., Magnusson, M., Malmström, E.-M., Melander, A., & Moritz, U. (1996). Postural and Symptomatic Improvement After Physiotherapy in Patients With Dizziness of Suspected Cervical Origin. *Archives of Physical Medicine Rehabilitation, 77*(9), 874-882. doi:<https://doi.org/10.1016/S0003-9993(96)90273-7>

Knapstad, M. K., Nordahl, S. H. G., & Goplen, F. K. (2019). Clinical characteristics in patients with cervicogenic dizziness: A systematic review. *Health Science Report, 2*(9). doi:<https://doi.org/10.1002/hsr2.134>

Kollén, L., Hörder, H., Möller, C., & Frändin, K. (2017). Physical functioning in older persons with dizziness: a population-based study. *Aging clinical and experimental Research, 29*, 197-205. doi:<http://doi.org/10.1007/s40520-016-0567-9>

Kristiansen, L., Magnussen, L. H., Juul-Kristensen, B., Mæland, S., Nordahl, S. H. G., Hovland, A., . . . Wilhelmsen, K. T. (2019). Feasibility of integrating vestibular rehabilitation and cognitive behaviour therapy for people with persistent dizziness. *Pilot and feasibility Studies*. doi:<https://doi.org/10.1186/s40814-019-0452-3>

Kuwabara, J., Kondo, M., Kabaya, K., & Watanabe, W. (2020). Acceptance and commitment therapy combined with vestibular rehabilitation for persistent postural-perceptual dizziness: A pilot study. *American Journal of Otolaryngology, 41*(6). doi:<https://doi.org/10.1016/j.amjoto.2020.102609>

Kwon, Y. H., & Ko, Y. M. (2017). Review on Physical Therapy for Patients with Vestibular Disorder. *The Journal of Korean Physical Therapy, 29*(6), 316-323. doi:<https://doi.org/10.18857/jkpt.2017.29.6.316>

Liu, X.-M., Pan, F.-M., Yong, Z.-Y., Ba, Z.-Y., Wang, S.-J., Liu, Z., . . . Wu, D.-S. (2017). Does the longus colli have an effect on cervical vertigo?: A retrospective study of 116 patients. *Medicine (Baltimore), 96*(12), e6365. doi:<http://doi.org/10.1097/MD.0000000000006365>

Lopes, A. L., Lemos, S. M., Figueiredo, P. H., & Santos, J. N. (2019). Impact of lian gong on the quality of life of individuals with dizziness in primary care. *Revista De Saude Publica, 53*(73). doi:<http://dx.doi.org/10.11606/s1518-8787.2019053001234>

Malaktaris, A., Lemons, P., Lynn, S. P., & Condons, l. (2014). Chilling Out. Meditation, Relaxation and Yoga. In *Health, Happiness, and Well-Being: Better Living Through Psychological Science*: Sage Publications.

Malmström, E.-M., Karlberg, M., Fransson, P.-A., Lindbladh, J., & Magnusson, M. (2009). Cervical proprioception is sufficient for head orientation after bilateral vestibular loss. *European Journal of Applied Physiology, 107*(1), 73-81. doi:<https://doi.org/10.1007/s00421-009-1097-3>

Meldrum, D., Herdman, S., Vance, R., Murray, D., Malone, K., Duffy, D., . . . McConn-Walsh, R. (2015). Effectiveness of conventional versus virtual reality-based balance exercises in vestibular rehabilitation for unilateral peripheral vestibular loss: results of a randomized controlled trial. *Archives of Physical Medicine and Rehabilitation, 96*(7), 1319‐1328.e1311. doi:<http://doi.org/10.1016/j.apmr.2015.02.032>

Murdin, L., & Harrop-Griffiths, K. (2021). Dizziness—The Audiovestibular Perspective. *Postural Tachycardia Syndrome*. doi:<https://doi.org/10.1007/978-3-030-54165-1_19>

Nada, E. H., Ibraheem, O. A., & Hassaan, M. R. (2019). Vestibular rehabilitation therapy outcomes in patients with persistent postural-perceptual dizziness. *Annals of Otology, Rhinology & Laryngology*. doi:<https://doi.org/10.1177/0003489418823017>

Odderson, I. R. (2020). A new treatment for cervical vertigo with botulinum toxin. *Journal of Clinical Neuroscience, 73)*, 316-317. doi:<https://doi.org/10.1016/j.jocn.2020.01.016>

Odkvist, I., & Odkvist, L. M. (1988). Physiotherapy in vertigo. *Acta Oto-Laryngologica, Supplement, 455*(455), 74-76.

Pereira, C. M. M., Pinheiro do Vale, J. d. S., de Oliveira, W. P., Pinto, D. d. S., Cal, R. V. R., de Azevedo, Y. J., & Bahmad, F. J. (2020). Aquatic physiotherapy: a vestibular rehabilitation option. *Brazilian Journal of Otorhinolaryngology*, 7. doi:<https://doi.org/10.1016/j.bjorl.2019.12.003>

Piker, E. G., Jacobson, G. P., & Newman, C. W. (2020). Assessing Dizziness-Related Quality of Life. In G. P. Jacobsen, N. T. Shepard, K. Barin, K. Janky, & D. L. McCaslin (Eds.), *Balance Function Assessment and Management* (3 ed., pp. 717): Plural publishing.

Reid, S. A., Callister, R., Katekar, M. G., & Rivett, D. A. (2014). Effects of cervical spine manual therapy on range of motion, head repositioning, and balance in participants with cervicogenic dizziness: a randomized controlled trial. *Archives of Physical Medicine & Rehabilitation, 95*(9), 1603-1612. doi:<http://doi.org/10.1016/j.apmr.2014.04.009>

Ricci, N. A., Aratani, M. C., Caovilla, H. H., & Ganança, F. F. (2012). Effects of conventional versus multimodal vestibular rehabilitation on functional capacity and balance control in older people with chronic dizziness from vestibular disorders: design of a randomized clinical tria. *Trials*. doi:<https://doi.org/10.1186/1745-6215-13-246>

Saman, Y., Mclellan, L., Mckenna, L., Dutia, M. B., Obholzer, R., Libby, G., . . . Bamiou, D.-E. (2016). State anxiety subjective imbalance and handicap in vestibular schwannoma. *Frontiers in Neurology, 7*. doi:<https://doi.org/10.3389/fneur.2016.00101>

Schenk, R. P. T., Coons, L. B., Bennett, S., & Huijbregts, P. A. (2006). Cervicogenic dizziness: a case report illustrating orthopaedic manual and vestibular physical therapy comanagement. *Journal of Manual & Manipulative Therapy, 14*(3), 56E-58E. doi:<http://doi.org/10.1179/jmt.2006.14.3.56E>

Scherer, H. (1985). Neck-induced vertigo. *Archives of Oto-Rhino-Laryngology - Supplement, 2*, 107-124. Retrieved from <http://ovidsp.ovid.com/ovidweb.cgi?T=JS&CSC=Y&NEWS=N&PAGE=fulltext&D=med2&AN=3868980>

Schmid, D. A., Allum, J. H., Sleptsova, M., Gross, S., Gaab, J., Welge-Lüssen, A., . . . Langewitz, W. (2018). *Effects of a program of cognitive-behavioural group therapy, vestibular rehabilitation, and psychoeducational explanations on patients with dizziness and no quantified balance deficit, compared to patients with dizziness and a quantified balance deficit* (Vol. 105): Elsevier.

Sezier, A. (2016). *The Experience of Chronic Subjective Dizziness: A Qualitative Exploratory Study.* (Master thesis). Auckland University of Technology, Retrieved from <http://openrepository.aut.ac.nz/handle/10292/10105>

Sezier, A., Saywell, N., Terry, G., Taylor, D., & Kayes, N. (2019). Working-age adults’ perspectives on living with persistent postural-perceptual dizziness: a qualitative exploratory study. *BMJ Open, 9*(4), e024326. doi:<http://doi.org/10.1136/bmjopen-2018-024326>

Stone, J., & Carson, A. (2017). An integrated approach to other functional neurological symptoms and related disorders. In B. Dworetzky & G. Baslet (Eds.), *Psychogenic Nonepileptic Seizures: Toward the Integration of Care*: Oxford University Press.

Sveinsdóttir, L. B. (2014). *Cervicogenic dizziness. The effect of manual therapy and deep neck flexor training.* (Master thesis). University of Bergen, Bergen. Retrieved from <https://bora.uib.no/bora-xmlui/handle/1956/9139>

Szirmai, A., Maihoub, S., Molnar, A., Fent, Z., Tamas, L., & Polony, G. (2020). Effect of the stapedius and tensor tympani muscles tenotomy on the quality of life of patients suffering from Meniere's disease. *Orvosi Hetilap, 161*(5), 177-182. doi:<https://doi.org/10.1556/650.2020.31634>

Talewar, K. K., Cassidy, E., & McIntyre, A. (2018). Living with Ménière's disease: an interpretative phenomenological analysis. *Disability and Rehabilitation, 42*(12), 1714-1726. doi:<https://doi.org/10.1080/09638288.2018.1534994>

Thomas, L., & Treleaven, J. (2020). Should we abandon positional testing for vertebrobasilar insufficiency? *Musculoskeletal Science and Practice, 46*. doi:<https://doi.org/10.1016/j.msksp.2019.102095>

Thomas, Z., & Smith, G. (2020). *Fear Avoidance Beliefs and Misdiagnosis in a 16-year-old Patient with Neck Pain, Headaches, and Dizziness: A Case Report*. Paper presented at the digitalcommons.ithaca.edu. <https://digitalcommons.ithaca.edu/cgi/viewcontent.cgi?article=1805&context=whalen>

Thoomes-de Graaf, M., & Thoomes, E. (2016). Focus Dizziness Is cervicogenic Dizziness an independent musculoskeletal Entity? *Manuelle Therapie, 20*(3), 109-115. doi:<https://doi.org/10.1055/s-0042-108661>

Tjell, C., & Iglebekk, W. (2012). Postural mismatch in musculoskeletal disorders. In M. Alricsson (Ed.), *Musculoskeletal Disorders*.

Tjernström, F., Zur, O., & Jahn, K. (2016). Current concepts and future approaches to vestibular rehabilitation. *Journal of Neurology, 263*, 65-70. doi:<https://doi.org/10.1007/s00415-015-7914-1>

Tramontano, M., Consorti, G., Morone, G., & Lunghi, C. (2020). Vertigo and Balance Disorders–The Role of Osteopathic Manipulative Treatment: A Systematic Review. *Complementary Medicine Research*. doi:<https://doi.org/10.1159/000512673>

van de Berg, R., Widdershoven, J., Bisdorff, A., Evers, S., Wiener-Vacher, S., Cushing, S. L., . . . Lempert, T. (2021). Vestibular migraine and recurrent vertigo of childhood: Diagnostic criteria consensus document of the Classification Committee of Vestibular Disorders of the Bárány Society and the International Headache Society. *Journal of Vestibular Research, 31*, 1-9. doi:<http://doi.org/10.3233/VES-200003>

Walker, A., Kantaris, X., & Mary, C. (2018). Understanding therapeutic approaches to anxiety in vestibular rehabilitation: a qualitative study of specialist physiotherapists in the UK. *Disability and Rehabilitation, 40*(7), 829-835. doi:<https://doi.org/10.1080/09638288.2016.1277393>

Weeks, V. D., & Travell, J. (1955). Postural vertigo due to trigger areas in the sternocleidomastoid muscle. *Journal of Pediatrics, 47*(3), 315-327. doi:<http://doi.org/10.1016/s0022-3476(55)80006-6>

Whitney, S. L., Sparto, P. J., & Furman, J. M. (2020). Vestibular Rehabilitation and Factors That Can Affect Outcome. *Seminars in Neurology, 40*(1), 165-172. doi:<Http://doi.org/10.1055/s-0039-3402062>

Wilhelmsen, K. T. (2010). *Symptoms and signs in patients with long-lasting dizziness.* (Doctoral Doctoral thesis). University of Bergen, Retrieved from <http://bora.uib.no/handle/1956/4034>

Wilhelmsen, K. T., & Kvåle, A. (2014). Examination and Treatment of Patients With Unilateral Vestibular Damage, With Focus on the Musculoskeletal System: A Case Series. *Physical Therapy, 94*(7), 1024-1033. doi:<http://doi.org/10.2522/ptj.20130070>

Wurthmann, S., Holle-Lee, D., Obermann, M., Roesner, M., Nsaka, M., Scheffler, A., . . . Naegel, S. (2021). Reduced Vestibular Perception Thresholds in Persistent Postural-Perceptual Dizziness-A Cross-Sectional Study. *Research Square*. doi:<https://doi.org/10.21203/rs.3.rs-142976/v1>

Yao, M., Tang, Z. Y., Cui, X. J., Sun, Y. L., Ye, X. L., Wang, P., . . . et al. (2020). Shi-Style Cervical Mobilizations Versus Massage for Cervical Vertigo: a Multicenter, Randomized, Controlled Clinical Trial. *Journal of alternative and complementary medicine (New York, N.Y.), 26*(1), 58‐66. doi:<http://doi.org/10.1089/acm.2019.0113>

Zaleski-King, A. (2020). *Oculomotor and Perceptual Measures of Visual Motion Sensitivity in Individuals with Chronic Dizziness.* (Doctoral Dissertation). Gallaudet University,

Zhang, Y. H., Liu, C. R., & Fu, B. Z. (2014). Thirty-three cases of positional vertigo treated by acupuncture at neck muscle trigger point. *Zhongguo Zhenjiu, 34*(3), 235-236. Retrieved from <https://pubmed.ncbi.nlm.nih.gov/24843961/>

Zur, O., Schoen, G., Dickstein, R., Feldman, J., Berner, Y., Dannenbaum, E., & Fung, J. (2015). Anxiety among individuals with visual vertigo and vestibulopathy. *Disability and Rehabilitation, 37*(23), 2197-2202. doi:<https://doi.org/10.3109/09638288.2014.1002577>
